# Characterization of an AsnC/Lrp-family transcriptional regulator in *Herbaspirillum rubrisubalbicans* M1: linking plant interaction, nitrogen response and PHB metabolism

**DOI:** 10.3389/fmicb.2026.1792436

**Published:** 2026-06-04

**Authors:** Roxana Beatriz Ribeiro Chaves, Luis Paulo Silveira Alves, Bruno Thiago de Lima Nichio, Thalita Regina Tuleski, Valter Antônio Baura, Marcelo Muller-Santos, Estevan Tomazini, André Sampaio, Emanuel Maltempi Souza, Rose Adele Monteiro

**Affiliations:** 1 Department of Biochemistry and Molecular Biology, Federal University of Paraná, Curitiba, Brazil; 2 Molecular Biology Institute of Paraná, Curitiba, Brazil; 3 Federation of Industries of the State of Paraná, Curitiba, Brazil

**Keywords:** AsnC/Lrp transcriptional regulator, gene expression, *Herbaspirillum rubrisubalbicans*, motility, biofilm formation, nitrogen limitation, PHB metabolism, root colonization

## Abstract

**Introduction:**

The mutation of *Hrubri_0242* in *Herbaspirillum rubrisubalbicans* M1 altered the regulation of genes associated with polyhydroxybutyrate (PHB) metabolism, including *phaC1*, *phaP1, phaP2, phaZ1*, and *phaZ2*, disrupting the coordinated balance between biopolymer synthesis, stabilization, and degradation.

**Methods:**

Quantitative GC-FID analyses, fluorescence microscopy, and gene expression analysis were used to evaluate PHB metabolism, intracellular granule formation, and transcriptional responses under nitrogen-replete and nitrogen-limited conditions.

**Results:**

Quantitative GC-FID analyses revealed a dynamic accumulation–consumption cycle of PHB in the wild-type strain, whereas the hr0242:Tn5 mutant exhibited reduced production, impaired turnover, and earlier depletion of intracellular reserves. Although both strains synthesized PHB under nitrogen-replete and nitrogen-limited conditions, accumulation was less sustained in the mutant, indicating compromised storage capacity. Fluorescence microscopy confirmed the presence of intracellular granules in both strains, with lower intensity and frequency in the mutant. Gene expression analysis revealed coordinated regulation of genes involved in PHB metabolism in the wild type according to nitrogen availability, whereas the mutant exhibited reduced *phaP1* expression, compensatory activation of *phaP2*, and increased expression of depolymerases, resulting in enhanced polymer turnover. In addition to metabolic effects, disruption of *Hrubri_0242* significantly reduced swarming motility, indicating broader physiological consequences.

**Discussion:**

Together, these findings demonstrate that *Hrubri_0242* functions as an integrative transcriptional regulator linking nitrogen-responsive gene expression to PHB metabolic homeostasis and motility, thereby contributing to physiological adaptation in *H. rubrisubalbicans* M1.

## 1 Introduction

The genus *Herbaspirillum*, belonging to the β class of the phylum Proteobacteria, comprises Gram-negative bacteria with spiral, vibrioid, or helical morphology, capable of motility in the presence of oxygen. Cells can present one to three flagella at one or both poles (Baldani et al., 1986, 1992). This genus includes microorganisms with diverse lifestyles, encompassing free-living forms in soil and water, as well as human pathogens (Berg et al., 2005).

Among endophytic diazotrophs, prominent genera include *Rhizobium* (Richardson et al., 2009), *Herbaspirillum*, *Azospirillum*, *Gluconacetobacter*, *Burkholderia*, and *Azoarcus* (Baldani et al., 1997), cyanobacteria and actinomycetes (Postgate, 1982). These microorganisms establish beneficial interactions with plants, promoting plant growth through biological nitrogen fixation and increasing the availability of essential nutrients such as phosphorus, sulfur, iron, and copper (Brencic and Winans, 2005).

In the agricultural context, *Herbaspirillum seropedicae* and *Hrubri_*M1 have been extensively studied due to their ability to establish endophytic associations with economically important grasses, such as maize (*Zea mays*), sugarcane (*Saccharum officinarum*), and rice (*Oryza sativa*) (Baldani et al., 1986; Boddey et al., 1995; James et al., 1999; Elbeltagy et al., 2001). Studies have shown that inoculation of *H. seropedicae* strains in rice can result in nitrogen fixation rates of up to 54%, leading to significant increases in plant growth (Baldani et al., 2000; Gyaneshwar et al., 2002). Additional data indicated that *H. seropedicae* expresses *nif* genes during colonization of rice roots and aerial parts, demonstrating its capacity for *in planta* nitrogen fixation (James et al., 2002; Roncato-Maccari et al., 2003).

Among species of the genus *Herbaspirillum*, *H. rubrisubalbicans* M1 stands out for its ecological versatility, acting either as a plant growth promoter or as an opportunistic pathogen in susceptible crops, such as sorghum and sugarcane, causing diseases like red stripe and mottled stripe, respectively (Olivares et al., 1997). The initial interaction of this bacterium with grasses occurs through adhesion to the root surface, followed by penetration into vascular tissues and colonization of intercellular spaces in the xylem and parenchyma (Monteiro et al., 2012). Previous transcriptomic analyses conducted in our research group indicated differential expression of genes associated with nitrogen metabolism, polysaccharide biosynthesis, and adaptation to the plant environment during interaction with sorghum, suggesting a complex regulatory network in *H. rubrisubalbicans*. Among these, *Hrubri_0242*, encoding a putative AsnC/Lrp-family transcriptional regulator, was significantly upregulated under plant interaction conditions, emerging as a candidate regulator potentially involved in coordinating these responses. Based on this observation, *Hrubri_0242* was selected for further functional characterization.

Transcriptional regulation plays a central role in bacterial adaptation to dynamic environments, such as the interior of plant tissues. Regulatory proteins act by activating or repressing gene expression in response to external signals, often through interaction with small effector molecules (Huffman and Brennan, 2002). Many of these regulatory factors belong to the helix-turn-helix (HTH) superfamily, which includes structural domains widely distributed among prokaryotes, such as winged helix (WH) and β-ribbon motifs (Brennan, 1993). Among global regulators, the AsnC family stands out, controlling the expression of genes related to cellular metabolism and responses to variations in nitrogen availability (Willins et al., 1991; Thaw et al., 2006). The Lrp regulator, for example, modulates the expression of genes associated with amino acid biosynthesis, nutrient transport, and nucleotide transhydrogenation in *Escherichia coli*, highlighting its role in maintaining cellular homeostasis (Calvo and Matthews, 1994; Yang et al., 2002).

Although transcriptional regulation has been extensively studied in other nitrogen-fixing bacteria, little is known about the regulatory mechanisms that coordinate metabolic adaptation in *H. rubrisubalbicans*, particularly those mediated by AsnC/Lrp-type regulators. Processes potentially modulated by these regulators include metabolic pathways associated with motility, adhesion, and the accumulation of storage polymers such as polyhydroxybutyrate (PHB), which plays a key role in stress adaptation and interaction with host plants.

Given the relevance of transcriptional regulators in bacterial adaptation, understanding the mechanisms controlling gene expression in *H. rubrisubalbicans* can provide valuable insights into its interaction with host plants and its biotechnological potential. Therefore, this study aims to characterize the transcriptional regulator belonging to the AsnC/Lrp family and evaluate its influence on gene expression and PHB metabolism in *H. rubrisubalbicans* under different nitrogen conditions

## 2 Materials and methods

### 2.1 Bioinformatics analysis

To select bacteria for comparative analysis, the amino acid sequence of *Hrubri_0242* was submitted to BLASTp^1^ against the NCBI Reference Sequence Database (RefSeq), using default parameters (E-value ≤ 1 × 10^–5^, query coverage ≥ 70%). From the top 100 hits, sequences were filtered to retain those with ≥30% identity covering the full-length protein. Redundant sequences (>95% identity) were removed.

The finalized alignment set was processed via ClustalW^2^ and comprised: (i) the closest homologs within the *Herbaspirillum* genus to evaluate intra-genus conservation; and (ii) representative sequences from bacterial genera phylogenetically distinct from *Herbaspirillum*, included to evaluate intergeneric diversity and conservation of domain architecture across broader taxonomic distances. Furthermore, the *Hrubri_0242* protein sequence was screened against the CATCHDB database^3^ to identify functional domains; those yielding the most robust scores (lowest e-values) were prioritized for downstream analysis. This dual-criterion approach enabled a comprehensive assessment of conservation at both the genus level and the broader domain-level homology within the bacterial domain.

*In silico* analyses allowed the determination of possible functions and metabolic pathways potentially involved by comparing the genes with those from bacteria of the same or different genera as *Herbaspirillum*. Nucleotide and amino acid (aa) sequences were used to identify families and domains, perform alignments and identity analyses, and determine gene positions as well as their clusters.

The gene cluster was determined based on grouping provided by the online KEGG (Kyoto Encyclopedia of Genes and Genomes) platform.^4^

Gene positions within the genome were determined from nucleotide sequences in GenBank (.gb) format using Artemis^5^, which allowed the assessment of potential promoters, operons, and the functions of each analyzed gene.

### 2.2 Bacterial strain maintenance

*Escherichia coli* cultures were grown in Luria-Bertani (LB) medium, composed of tryptone (10 g/L), yeast extract (5 g/L), and NaCl (10 g/L), under agitation at 120 rpm and 37 °C. For solid media, 15 g/L agar was added to LB. All media were autoclaved at 120 °C and 1.2 atm for 20 min before use.

*Herbaspirillum rubrisubalbicans* M1 strains were grown in NFbHPN-Malate liquid medium containing 20 mmol/L NH_4_Cl as a nitrogen source at 30 °C under agitation at 120 rpm for 18–24 h. The medium contained MgSO_4_⋅7HO_2_O (0.2 g/L), NaCl (0.1 g/L), potassium malate (5 g/L), CaCl_2_ (0.02 g/L), nitrilotriacetic acid (0.056 g/L), FeSO_4_⋅7H_2_O (0.02 g/L), biotin (0.0001 g/L), 10 mL of oligonutrient solution, and 50 mL of phosphate solution per liter. The oligonutrient solution contained Na_2_MoO_4_⋅2H_2_O (1 g/L), MnSO_4_⋅H_2_O (1.175 g/L), H_3_BO_3_ (1.4 g/L), CuSO_4_⋅5H_2_O (0.04 g/L), and ZnSO_4_⋅7H_2_O (0.12 g/L), completed with distilled water to 1 L. Solid and semi-solid media contained 1.5% and 0.175% (w/v) agar, respectively.

Antibiotics used included tetracycline, kanamycin, and streptomycin, with strain-specific concentrations for *E. coli* and *H. rubrisubalbicans.* Stock solutions were prepared in autoclaved distilled water or 70% ethanol for tetracycline and stored at 4 °C.

### 2.3 Generation of *H. rubrisubalbicans* M1 mutant strains

Mutant strains were generated using primers (forward 5′–CGGGATCCTTAACCTCCCAGCCCTTGCCT–3′, reverse 3′–GAATTCCCATATGTTAATGATGCCGATTTCCTCCTCC–5′) designed from the *Hrubri_0242* gene sequence of *H. rubrisubalbicans* M1. The primers were amplified and directly ligated into the pSUP202 vector. Ligation-based cloning was performed by introducing the ligation mixture into *E. coli* S17 via chemical transformation using the CaCl_2_ method described by Chan et al. (2013). Following transformation, positive clones were confirmed by alkaline lysis plasmid DNA extraction (Sambrook et al., 1989), restriction digestion with *Eco*RI (FERMENTAS^®^), and sequencing.

The insertion was disrupted using an *in vitro* transposition technique (EZ:TN <*KM*−1>, Epicentre Laboratories), conferring kanamycin resistance (Km), which was verified by *Eco*RI restriction. Transposon-confirmed constructs were conjugated into *H. rubrisubalbicans* M1.

Mutants resulting from single homologous recombination were obtained through negative selection using tetracycline (Tc) and kanamycin (Km). Mutant confirmation was performed by PCR using a gene-specific primer, followed by analysis of amplicon size by agarose gel electrophoresis. Cassette insertion was confirmed by PCR (Kocher et al., 1989) and cleavage verified by 1% agarose gel electrophoresis (TBE 1X). PCR reactions used 10 pmol primers Y1 and Y3 for S17 clones and primers specific to the target gene for mutant confirmation. Approximately 10 ng of genomic DNA, 0.2 mM dNTPs, 1.5 mM MgCl^2^, and 1 U Taq DNA polymerase were used in a 50 μL reaction with the following cycling parameters: 1 cycle at 95 °C for 1 min, 30 cycles at 94 °C for 45 s, 50 °C–60 °C for 45 s, 72 °C for 1 min 30 s, and a final extension at 72 °C for 10 min.

### 2.4 Bacterial conjugation

Donor *E. coli* S17.1 and recipient strains were cultured in appropriate medium for 16 h at suitable temperatures under constant agitation. Cultures were reinoculated at 1/50 (recipient) and 1/100 (donor) in antibiotic-free medium for 6 h (recipient) and 3 h (donor).

Five microliters of the donor strain were mixed with 50 μL of the recipient strain. The mixture was deposited as a drop onto NFbHPN plates supplemented with LA medium (3:1 ratio) and incubated at 30 °C for ∼16 h. Cells were resuspended in 1 mL liquid NFbHPN, and 100 μL was plated on solid NFbHPN containing antibiotics for transconjugant selection. Plates were incubated at 30 °C until colonies were visible.

### 2.5 Growth curve analysis

Strains were pre-cultured overnight at 30 °C and 120 rpm in 3 mL NFbHPN-malate medium supplemented with 20 mM NH_4_Cl and antibiotics. Cultures were diluted to an initial ODW_595_ of 0.01 (approximately 1 × 10^5^ CFU mL^–1^) to initiate growth assays. Triplicate cultures were grown in 125 mL Erlenmeyer flasks containing 25 mL NFbHPN-malate medium with 20 mM NH_4_Cl, without antibiotics, at 30 °C and 120 rpm.

Bacterial growth was monitored by measuring optical density at 595 nm (OD_595_) every 2 h for 12 h using a BioTek Elx800 microplate reader. Growth rate (μ) was determined from the exponential phase by plotting the natural logarithm of OD values and fitting the equation ln(OD_595_)t = μt + ln(OD_595_)0, as described by Doran (2004). Doubling time (t_d) was calculated using the equation t_d = ln2/μ.

### 2.6 Motility assays

For motility assays, strains were cultured in NFbHPN-malate medium with 20 mM NH_4_Cl at 30 °C and 120 rpm until OD_595_nm ∼1. Inocula were diluted to OD_595_nm 1 (∼108 CFU mL^–1^) and 10 μL applied to the center of NFbHPN-malate semi-solid plates (0.3% agar, 20 mM N). Plates were incubated at 30 °C for 48 h, and motility halo radii were measured in quintuplicate at three points from the center using ImageJ.

### 2.7 Biofilm formation on glass fiber filters

*H. rubrisubalbicans* M1 strains were cultured overnight to OD_595_nm 1 in NFbHP-malate (20 mM NH_4_Cl) at 30 °C and 120 rpm. Cells were diluted to OD_595_nm 0.01 (∼10^6^ CFU mL^–1^), and 10 mL was added to sterilized 50 mL glass tubes containing fiberglass filters, incubated at 30 °C and 120 rpm for 6, 12, and 24 h. Non-inoculated filters served as negative controls. Planktonic cells were removed, filters washed with 10 mL 0.9% NaCl for 10 min at 50 rpm, stained with 200 μL 0.1% crystal violet for 5 min, washed five times, and the retained dye solubilized in 1 mL ethanol and read at 540 nm.

### 2.8 *In planta* assays

Sorghum (*Sorghum bicolor* BR 310) and maize (*Zea mays* 30A37PW) seedlings were surface-sterilized with 70% ethanol for 5 min and 1% sodium hypochlorite for 5 min, rinsed with sterile Milli-Q water, and germinated on water-agar at 30 °C in the dark for 48 h. Seedlings were inoculated with *H. rubrisubalbicans* M1 (wild-type and mutant) at 105 CFU mL^–1^ for 30 min, rinsed, and transferred to Plant Medium tubes with sterile polypropylene beads, incubated at 28 °C with a 12 h photoperiod. Initial bacterial concentration was confirmed by plating on solid NFbHPN-malate. Plant Medium composition per liter: MgSO_4_⋅H_2_O 0.2 g, NaCl 0.1 g, CaCl_2_⋅2H_2_O 0.026 g, 1 mL microelement solution, 1 mL Fe-EDTA (1.64%), K_2_HPO_4_ 1.5 g, KH_2_PO_4_ 1.5 g. Microelements: Na_2_MoO_4_⋅2H_2_O 1 g, MnSO_4_⋅H_2_O 1.175 g, H_3_BO_3_ 1.4 g, CuSO4⋅5H_2_O 0.04 g, ZnSO4⋅7H_2_O 0.12 g.

### 2.9 Root adhesion assays

After inoculation, roots were washed in sterile saline, cut, placed in pre-weighed 1.5 mL centrifuge tubes, vortexed 1 min to detach adhered cells, serially diluted, and plated using the microdrop technique on NFbHPN-malate solid medium without antibiotics. Plates were incubated at 30 °C for 48 h. Negative controls consisted of seedlings incubated in sterile saline. Data were expressed as CFU/g root.

### 2.10 Competition assays for sorgum root colonization

Mutant and wild-type strains were co-inoculated on sorgum roots at a 1:1 ratio, maintaining 105 CFU mL^–1^ per plant. CFU determination and antibiotic resistance were used to distinguish strains.

### 2.11 PHB quantification by gas chromatography

Bacterial PHB was quantified by acidic methanolysis followed by GC-FID. Lyophilized bacteria (5–10 mg) were incubated with 2 mL chloroform and 2 mL methanol containing 15% sulfuric acid and 0.5 mg benzoic acid as an internal standard at 100 °C for 3.5 h. After cooling, 1 mL water was added, vortexed, and the organic phase containing methyl 3-hydroxybutyrate (Me-3-HB) was dried with Na2SO4 and analyzed on a Varian 450 GC with a CP-Sil-5 CB column (10 m × 0.53 mm ID), using argon at 0.9 mL/min. Injector and detector temperatures were 250 °C and 275 °C, respectively. Oven program: 50 °C for 2 min, ramp to 110 °C at 20°C/min^–1^, then to 250 °C at 20^–1^. PHB content was normalized to lyophilized cell weight and expressed as% PHB/dry cell weight.

### 2.12 Fluorescence and transmission electron microscopy

Polyhydroxybutyrate granules were visualized using Nile Red staining. Cultures were centrifuged, resuspended in 30% ethanol (PBS), stained with 3 μL of 1.6 mM Nile Red, and observed with an Axio Imager Z2 microscope. For TEM, *H. seropedicae* cells were fixed with Karnovsky, post-fixed with OsO4, embedded in Epon 812, stained with uranyl acetate and lead citrate, and visualized on a JEOL-JEM 1200 EX II.

### 2.13 mRNA quantification by RT-qPCR

Gene expression was analyzed by reverse transcription quantitative PCR (RT-qPCR). Strains *Hrubri_*M1 and hr0242:Tn5 were grown in nitrogen-deficient medium (−N, 5 mM) for 24 h. Total RNA was extracted with TRIzol (Invitrogen) and treated with DNase I (Thermo Fisher Scientific). RNA integrity and concentration were verified by spectrophotometry (NanoDrop 2000, Thermo Scientific) and agarose gel electrophoresis.

cDNA was synthesized from 1 μg of RNA using the High Capacity cDNA Reverse Transcription Kit (Applied Biosystems, Thermo Fisher Scientific, Waltham, MA, United States; Cat. No. 4368814), and diluted 1:60. RT-qPCR was performed using Power SYBR Green PCR Master Mix (Applied Biosystems) on a StepOne Plus Real-Time PCR System (Applied Biosystems).

Polyhydroxybutyrate metabolism genes in *H. rubrisubalbicans* M1 were: *phaC1* (*H.rubri_3020*), *phaP1* (*H.rubri_1525*), *phaP2* (*H.rubri_4717*), *phaZ1* (*H.rubri_0609*), *phaZ2* (*H.rubri_1511*), and *phaR*/*phbF* (*H.rubri_3018*).

Primers were designed using Primer Express 3.0 (Applied Biosystems) and NCBI tools, validated with standard curves (R^2^ ≥ 0.98; efficiency ≥ 90%) (Supplementary Table 1).

Expression was normalized using the *rpoN* reference gene, identified as most stable by geNorm. Relative expression was calculated using the Pfaffl method and confirmed by 2^–Δ^ ^Δ^ *^Ct^*. Reactions were performed in technical triplicate with three independent biological replicates. Results are presented as log10 values, with error bars representing the standard deviation of technical replicates.

## 3 Results

### 3.1 Bioinformatics analysis

**FIGURE 1:**
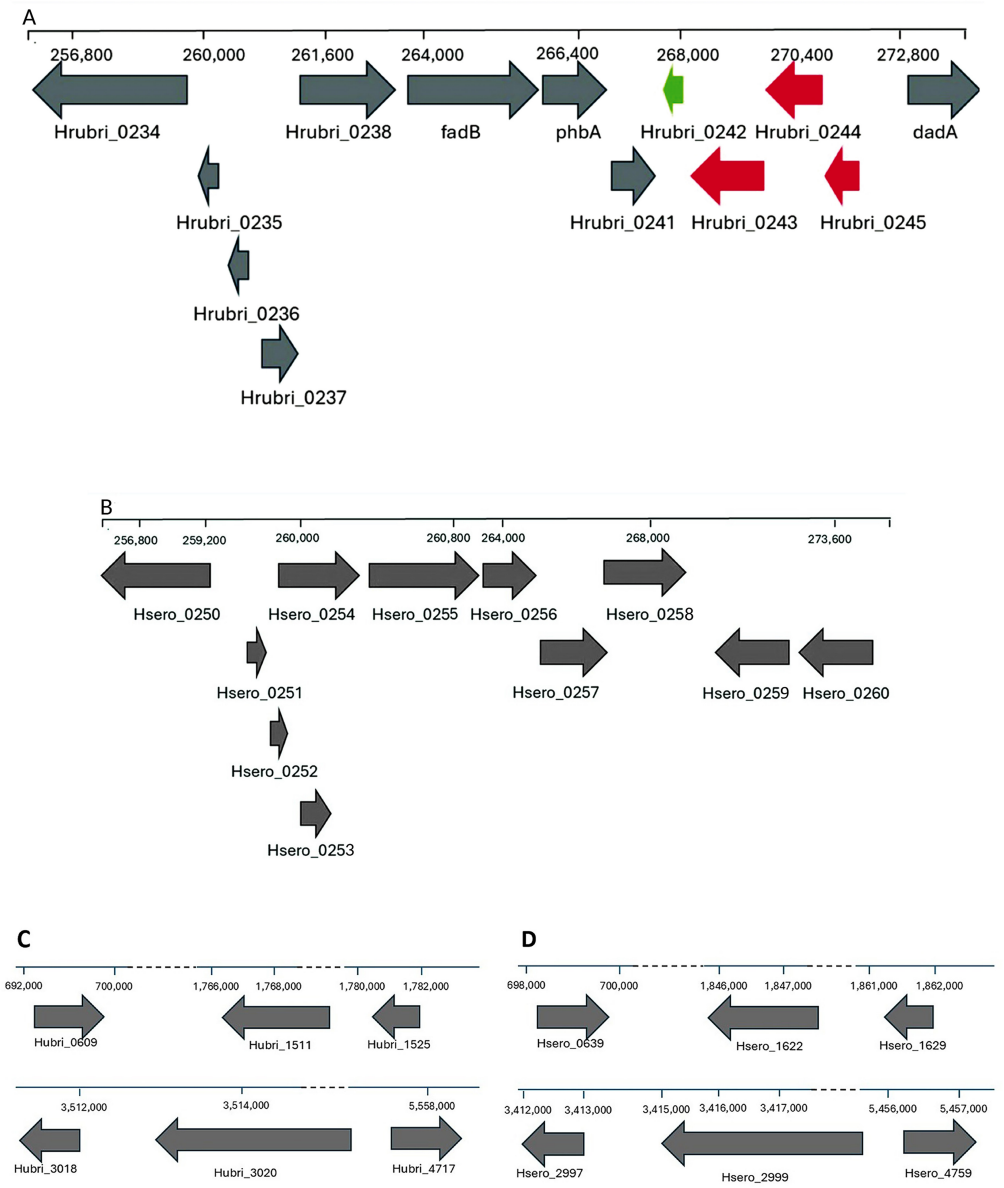
Comparative gene organization between *H. rubrisubalbicans* M1 and *H. seropedicae* SmR1. Comparative analysis of gene organization and polyhydroxybutyrate (PHB) metabolism-associated clusters in the two strains. Gene sequences were obtained from the Kyoto Encyclopedia of Genes and Genomes (KEGG) pathway database, and “*n*” represents the number of genes identified per cluster. **(A)** Gene cluster containing *Hrubri_0242* in *H. rubrisubalbicans* M1. **(B)** Corresponding genomic region in *H. seropedicae* SmR1, in which the operon is absent. **(C)** PHB metabolism genes in *H. rubrisubalbicans* M1. **(D)** PHB metabolism genes in *H. seropedicae* SmR1. Arrows indicate genes and their transcriptional orientation. The *Hrubri_0242* gene (AsnC/Lrp family transcriptional regulator) is highlighted in green. Genes shown in red belong to the same operon as *Hrubri_0242*, and genes in gray represent other adjacent ORFs in the genomic cluster. Comparison between **(C)** and **(D)** demonstrates the conserved organization of PHB metabolism genes. Scale bars indicate base pairs.

*Hrubri_0242* was selected for further study based on transcriptomic analyses conducted in our research group, which indicated that the gene was upregulated during interaction with sorghum. The encoded protein comprises 167 amino acids and was predicted to belong to the Lrp/AsnC family of transcriptional regulators. Genomic context analysis revealed that *Hrubri_0242* constitutes the final gene of an operon composed of Hrubri_0243 (4-aminobutyrate aminotransferase), *Hrubri_0244* (hydroxylysine kinase), and Hrubri_0245 (2-haloacid dehalogenase) (Figure 1A).

To evaluate conservation within the genus, the 10 closest homologs of *Hrubri_0242* within *Herbaspirillum* were identified using BLASTp. High sequence identity was observed among these homologs (Supplementary Figure 1), and all annotated sequences were consistently classified as members of the Lrp/AsnC family of transcriptional regulators. To assess conservation across broader phylogenetic distances, 21 additional homologs representing phylogenetically distinct bacterial genera spanning Proteobacteria and Actinobacteria were included in the analysis. Sequence similarities ranged from 64.07% to 88.02% (Supplementary Figure 2), indicating substantial conservation of this regulator across taxonomically distant bacterial lineages.

To further examine the conservation of the Lrp/AsnC structural architecture, the predicted domain boundaries of *Hrubri_0242* and its homologs were compiled in a comparative dataset including sequence length and the start and end positions of the Lrp core region, the helix–turn–helix (HTH) DNA-binding motif, and the AsnC regulatory domain. These data were obtained for homologs within the *Herbaspirillum* genus as well as for representatives of phylogenetically distinct bacterial taxa. The compiled dataset is provided in Supplementary Tables 2, 3, while the corresponding multiple sequence alignments are shown in Supplementary Figures 1, 2. Notably, all analyzed proteins displayed an identical length of 167 amino acids and a highly conserved domain organization, with the HTH DNA-binding motif located in the N-terminal region and the AsnC regulatory domain occupying the central–C-terminal portion of the protein. Minor variations in predicted domain boundaries were observed among taxa; however, the overall structural architecture characteristic of Lrp/AsnC transcriptional regulators was preserved across all analyzed homologs.

To further characterize the structural features of *Hrubri_0242*, the protein sequence was analyzed using the CATH database for domain identification. Two conserved domains characteristic of Lrp/AsnC regulators were detected. The N-terminal region contains a helix–turn–helix (HTH) DNA-binding domain (e-value: 2.7 × 10^–12^), showing structural similarity to the Escherichia coli template (PDB: 2L4A). The C-terminal region corresponds to the Lrp/AsnC effector-binding domain, also referred to as the RAM (Regulation of Amino acid Metabolism) domain (e-value: 6.9 × 10^–^8), consistent with the structural fold described for the *E. coli* Lrp protein (PDB: 2GQQ). The presence of these conserved domains, together with the observed sequence homology among bacterial homologs, supports the classification of *Hrubri_0242* as a member of the Lrp/AsnC family of transcriptional regulators.

*In silico* analyses using the Kyoto Encyclopedia of Genes and Genomes (KEGG) revealed that, unlike the other genes present in this operon, *Hrubri_0242* is absent from the genome of *H. seropedicae* SmR1 (Figure 1B). Examination of the genomic region surrounding *Hrubri_0242* in *H. rubrisubalbicans* M1 (Supplementary Table 4) further revealed that the organization of genes involved in polyhydroxybutyrate (PHB) metabolism is broadly conserved when compared with *H. seropedicae* SmR1 (Figure 1D), despite broader genomic differences between the strains (Figure 1C).

### 3.2 Obtention and characterization of bacterial strains

To investigate the function of the gene in *H. rubrisubalbicans* M1, a mutant strain was constructed via transposon-mediated gene disruption. The bacterial strains used in this study are listed in Table 1.

**TABLE 1:** Characteristics of the bacterial strains.

Strain	Characteristics	References
*E. coli* S17.1	*recA*, *hsdR* RP4-2-*Tc:Mu-Km:Tn7*	SIMON; PRIEFER; Simon et al., 1983
*H. rubrisubalbicans*		Pedrosa et al., 1997
M1	Wild-type strain, isolated from sorghum leaves	–
hr0242:Tn5	*H. rubrisubalbicans_*M1 harboring the plasmid 0242pSUP202-Tn, Tc^r^, Km^r^	This work

Km, kanamycin; Tc, tetracycline; and the superscript r, resistant. The designation “This work” refers to plasmids already obtained.

Initially, an internal fragment of approximately ∼500 bp of the gene was amplified by PCR and cloned directly into the pGEM-T vector (Promega) following the manufacturer’s protocol. Recombinant clones were confirmed by *Eco*RI digestion and sequencing.

Disruption of the *Hrubri_0242* gene was performed by *in vitro* transposition using the EZ-Tn5 < KAN-1 > system (Epicentre Laboratories), which inserts a kanamycin resistance cassette (Km^R^) at the cloning site. The transposon was inserted disrupting the central region of the coding sequence of the *Hrubri_0242* gene, resulting in loss of gene integrity and likely functional inactivation. The disrupted gene was subsequently subcloned into the conjugative vector pSUP202 (7,830 bp) for transfer into *H. rubrisubalbicans* M1. The recombinant construct (pSUP202-Hr0242:Tn5) was first transformed into *E. coli* Top10 and then transferred to the donor strain *E. coli* S17, which was used for conjugation with *H. rubrisubalbicans* M1.

Transconjugants were selected on solid NFbHPN medium containing kanamycin, and mutants generated by single homologous recombination were identified by their simultaneous resistance to kanamycin and tetracycline. Correct insertion of the Km^®^ cassette into the *Hrubri_0242* locus was confirmed by specific PCR, and strain identity was validated by 16S rDNA sequencing. The mutant strain hr0242:Tn5 showed no significant differences in growth pattern compared to the wild-type strain, indicating that gene deletion did not compromise cell viability under standard cultivation conditions.

### 3.3 Phenotypic characterization of wild-type and mutant strains of *H. rubrisubalbicans* M1: motility, biofilm formation, and competition in sorghum

**FIGURE 2:**
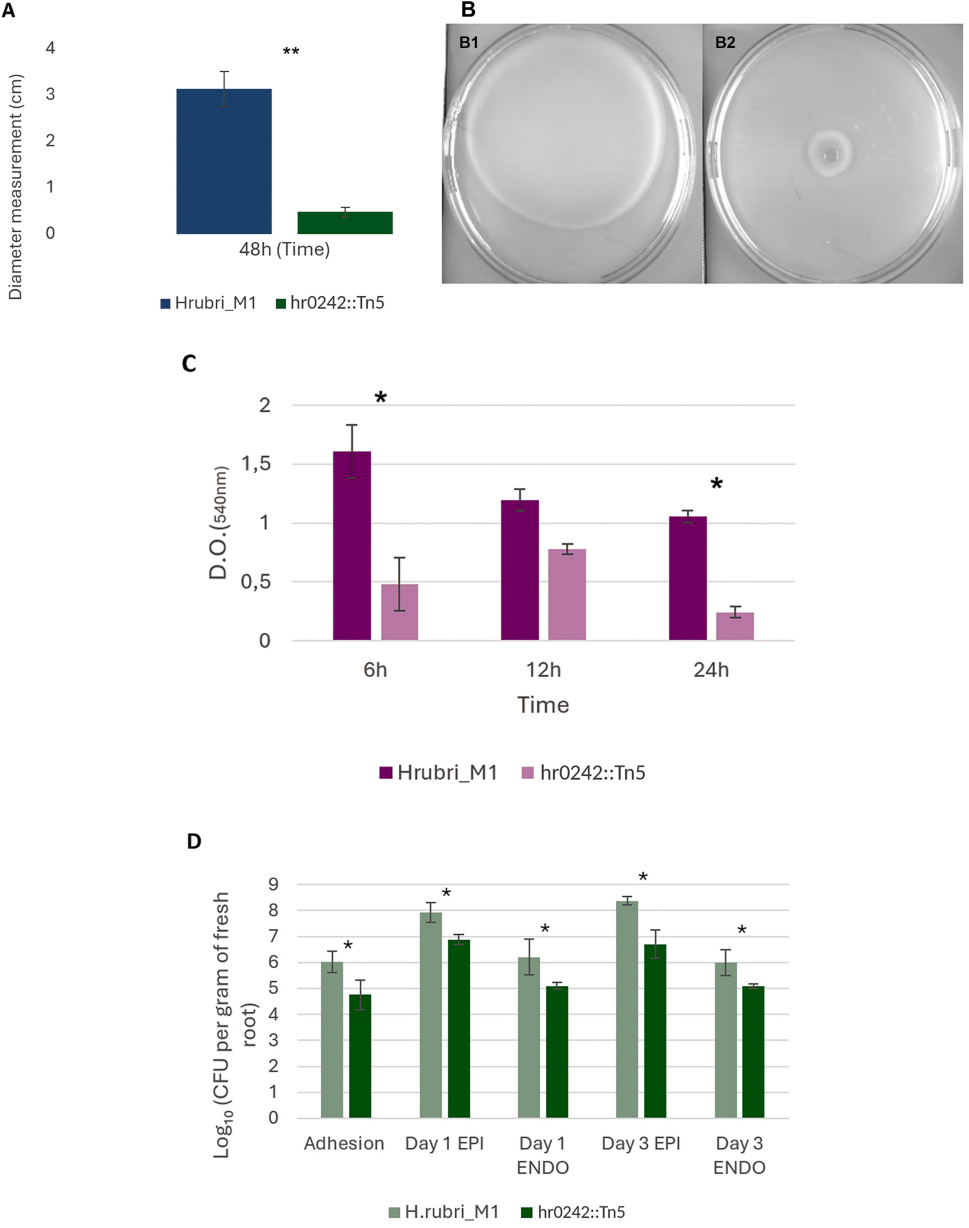
Phenotypic characterization of wild-type and mutant strains of *Hrubri*_M1. Comparative analyses of motility, biofilm formation, and root colonization were conducted to assess phenotypic differences between the wild-type and hr0242:Tn5 mutant strains. **(A)** Comparative graph of swarming motility between the wild-type strain (blue bar) and the mutant hr0242:Tn5 (green bar), obtained in quintuplicate from cultures adjusted to OD_595_ = 0.3. Analysis was performed after 24 h of incubation at 30 °C. **(B)** Motility assay on plates containing 0.3% NFb-malate medium supplemented with 20 mM nitrogen, showing the wild-type strain **(B1)** and mutant hr0242:Tn5 **(B2)** after 24 h of incubation at 30 °C. Experiments were performed in quintuplicate. **(C)** Biofilm formation assay on glass fiber supports using wild-type and mutant hr0242:Tn5 strains, performed in triplicate from cultures adjusted to OD_595_ = 0.01. Asterisks indicate *p* = 0.05. **(D)** Sorghum seedlings were inoculated with 105 cells of wild-type or mutant strains of *H. rubrisubalbicans* M1 without agitation for 40 min. The number of bacteria adhering to roots after washing was determined. Epiphytic and endophytic bacterial populations were quantified at 1 and 3 days after inoculation (d.a.i.). Results are expressed as the mean Log10 CFU per gram of fresh root ± standard deviation. Asterisks indicate statistically significant differences between wild-type and mutant strains (*t*-test; *p* = 0.05).

Phenotypic assays were performed to evaluate the impact of the *Hrubri_0242* mutation on motility, biofilm formation, and competitive root colonization (Figure 2). In swarming assays, the mutant strain hr0242:Tn5 exhibited reduced surface motility compared to the wild-type *H. rubrisubalbicans* M1, forming colonies with a smaller dispersion diameter after 24 h of incubation on semi-solid NFb-malate medium (Figures 2A, B). Quantitative analysis confirmed a consistent decrease in motility across all replicates.

Biofilm formation was analyzed on glass fiber supports over 24 h (Figure 2C). At all evaluated time points (6, 12, and 24 h), the mutant hr0242:Tn5 exhibited a significant reduction in biofilm biomass compared to the wild-type strain.

Competition assays between the wild-type strain and the mutant hr0242:Tn5 in sorghum seedlings showed that the mutant retained epiphytic and endophytic colonization capacity but performed worse than the wild-type strain (Figure 2D). Statistically significant differences were observed in both adhesion (epiphytic and endophytic) and colonization at 1 and 3 days post-inoculation. In contrast to studies in *Pseudomonas aeruginosa* (Sivakumar et al., 2020), interruption of *Hrubri_0242* in *H. rubrisubalbicans* M1 resulted in reduced competitive ability compared to the wild-type strain.

### 3.4 PHB quantification by gas chromatography

Polyhydroxybutyrate (PHB) functions as a carbon and energy storage compound in various bacteria and has been associated with adaptive responses to environmental fluctuations (Alves et al., 2016; Lee et al., 1995; Beeby et al., 2012). In plant-associated species, the synthesis and mobilization of this polymer are frequently linked to root colonization and nutritional stress responses. Considering that the ability of *Hrubri_*M1 to accumulate PHB had not been previously demonstrated, we quantified the biopolymer to assess whether disruption of the *H.rubri_0242* gene affects its production. PHB production was quantified by gas chromatography with flame ionization detection (GC-FID) in *Hrubri_*M1 and hr0242:Tn5 strains, cultured under two nitrogen availability conditions: high (+N, 20 mM NH_4_Cl) and low (−N, 5 mM NH_4_Cl). Cultures were monitored over 24 h, with sampling every 2 h. Growth curves were obtained by optical density (OD_595_) in three biological replicates, and PHB content was determined in technical duplicate for each time point, expressed as a percentage of cell dry weight (% cdw).

**FIGURE 3:**
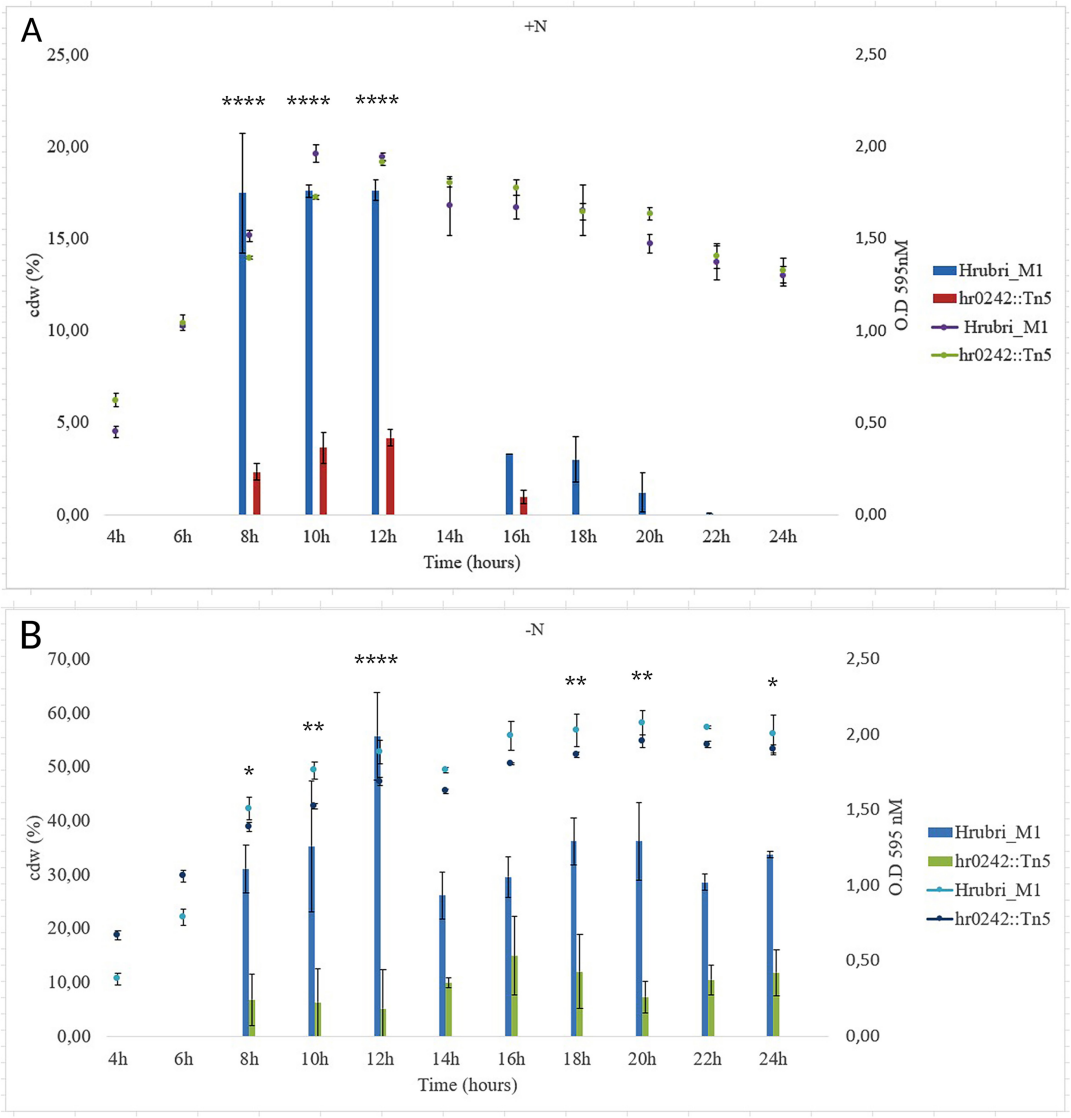
Growth and polyhydroxybutyrate (PHB) accumulation in *Hrubri_*M1 and the mutant strain hr0242:Tn5. Growth kinetics and PHB accumulation were evaluated under two nitrogen conditions. **(A)** Cultivation in medium containing 20 mM ammonium chloride (+N). **(B)** Cultivation in medium containing 5 mM ammonium chloride (–N). Bars represent PHB content (% cdw), and points indicate cell growth (OD_595_). Blue bars and blue points correspond to the wild-type strain *H. rubrisubalbicans* M1, while orange bars represent the mutant *Hrubri_0242*:Tn5 under +N conditions and green bars represent the mutant under –N conditions. Error bars represent standard deviation. Cultures were incubated at 30 °C with shaking at 120 rpm for 24 h. OD, optical density; cdw, cell dry weight. Statistical significance is indicated above the bars *p = 0.0315; **p = 0.0013; ****p < 0.0001.

Under high nitrogen conditions (+N, 20 mM) (Figure 3A), both strains exhibited a PHB accumulation pattern consistent with the transition to the stationary phase. Accumulation began between 6 and 8 h, reaching a peak at 12 h, when the wild-type strain accumulated ∼15%–18% PHB/cdw, whereas the mutant exhibited ∼3%–5% PHB/cdw. After 12 h, a continuous decrease in polymer levels was observed. PHB depletion occurred between 20 and 22 h in the wild-type strain and earlier, between 16 and 18 h, in the mutant hr0242:Tn5. This profile—accumulation during late exponential phase followed by mobilization at the transition to stationary phase—is typical of PHB-producing bacteria (Alves et al., 2016; Lee et al., 1995).

Under nitrogen limitation (−N, 5 mM) (Figure 3B), a distinct behavior was observed. Both strains initiated polymer synthesis after 8 h of cultivation, but the magnitude of accumulation was significantly higher in the wild-type strain (*p* ≤ 0.001). The peak occurred between 12 and 16 h, reaching ∼45%–55% PHB/cdw in *H. rubrisubalbicans* M1, while the mutant reached only 10%–15% PHB/cdw in the same period. After the peak, a gradual reduction was observed, with the parental strain maintaining residual levels of 25%–30% PHB/cdw at 24 h, whereas hr0242:Tn5 exhibited values below 10%, indicating lower stability and retention of the polymer over time. These results are consistent with previous studies in *H. seropedicae* SmR1, where nitrogen limitation promoted higher PHB accumulation (Alves et al., 2016).

Quantitative comparison between genotypes and conditions revealed three consistent patterns: (i) in both strains, nitrogen limitation promoted higher PHB accumulation compared to the +N condition; (ii) the wild-type strain displayed significantly higher PHB levels than the mutant at all time points after 8 h (*p* ≤ 0.001, Welch’s *t*-test); and (iii) the polymer consumption rate was more pronounced in the mutant, reflecting early mobilization of the compound during the initial stationary phase.

Unlike genes involved in PHB metabolism in *H. seropedicae* SmR1, whose mutation resulted in alterations of biopolymer metabolism (Batista et al., 2018), mutation of the *Hrubri_0242* gene led to reduced synthesis and accelerated degradation. These results indicate that *Hrubri_0242* may act at a distinct regulatory level, influencing PHB turnover in *H. rubrisubalbicans.*

Based on this differential PHB production and consumption profile between the wild-type and mutant strains, the next step was to investigate whether these phenotypic variations are associated with changes in the expression of genes involved in polymer metabolism, encompassing PHB synthesis, stabilization, and degradation enzymes.

### 3.5 Fluorescence microscopy reveals differences in PHB accumulation between strains under nitrogen limitation

**FIGURE 4:**
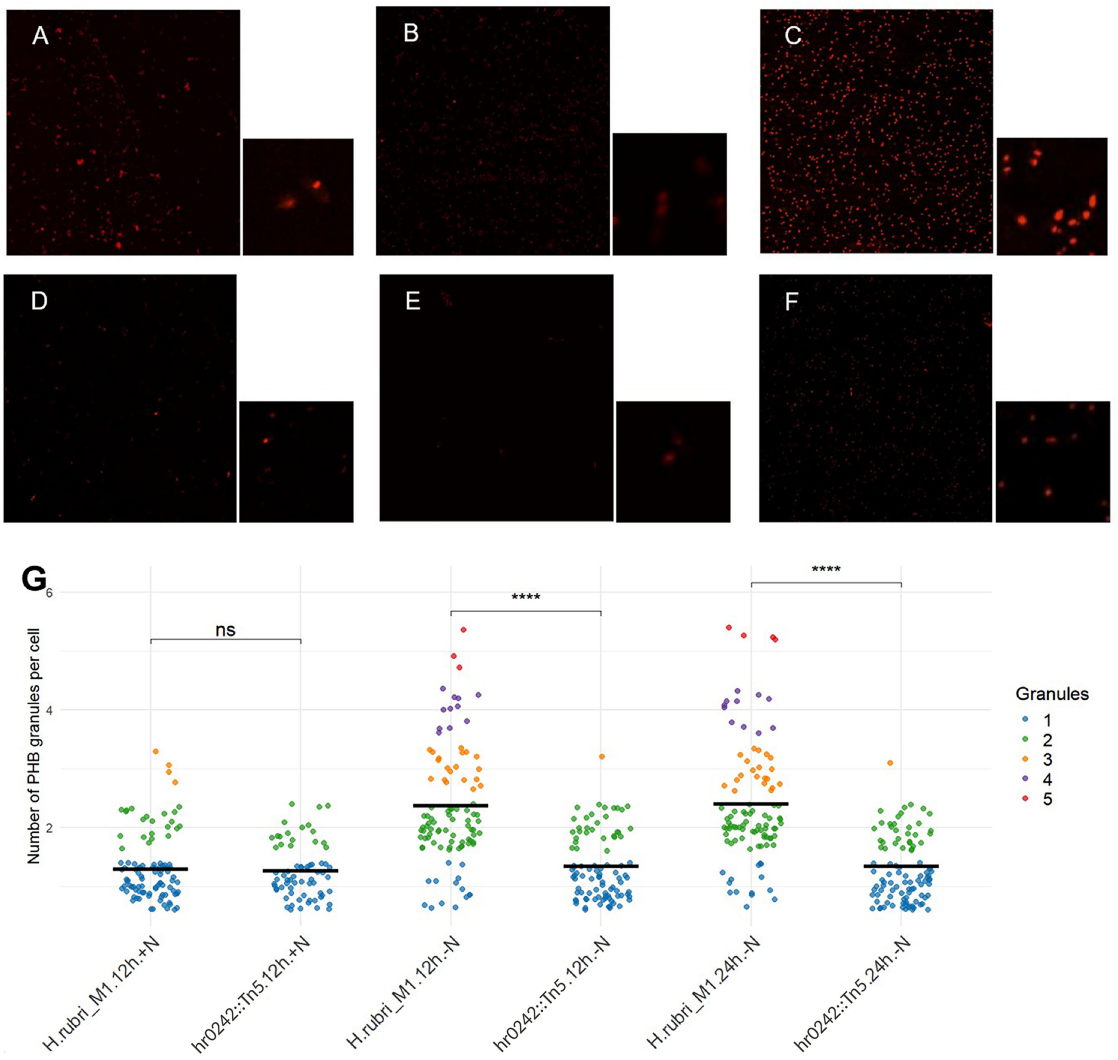
Fluorescence microscopy of *Hrubri_*M1 and the mutant strain hr0242:Tn5 stained with Nile Red. Polyhydroxybutyrate (PHB) granules were visualized in bacterial cells cultured for 12 and 24 h under nitrogen-supplemented (+N) or nitrogen-depleted (–N) conditions. Nile Red fluorescence highlights neutral lipid inclusions. **(A)** *H. rubrisubalbicans* M1, –N, 12 h. **(B)** *H. rubrisubalbicans* M1, +N, 12 h. **(C)** *H. rubrisubalbicans* M1, –N, 24 h. **(D)** hr0242:Tn5, –N, 12 h. **(E)** hr0242:Tn5,+N, 12 h. **(F)** hr0242:Tn5, –N, 24 h. Enlarged panels on the right highlight intracellular fluorescent granules. Nitrogen depletion favored PHB accumulation, particularly in the wild-type strain, while the mutant exhibited reduced fluorescence, indicating lower polymer accumulation. **(G)** Distribution of PHB granules per cell in *H. rubrisubalbicans* M1 and the mutant strain hr0242:Tn5 under nitrogen-sufficient (+N) and nitrogen-limiting (–N) conditions at 12 and 24 h. Stacked dot plots represent individual cells, with the color scale indicating the number of PHB granules (1–5) per cell. Each condition was analyzed by counting 100 cells. Comparisons between strains within the same condition (time and nitrogen availability) were performed using Fisher’s exact test. Statistical significance is indicated above the bars (****p < 0.0001; ns, not significant).

The number of polyhydroxybutyrate (PHB) granules per cell was determined by fluorescence microscopy after Nile Red staining to assess intracellular accumulation patterns in *H. rubrisubalbicans* M1 and hr0242:Tn5 strains under high nitrogen (+N, 20 mM) and low nitrogen (−N, 5 mM) conditions. Images obtained after 12 and 24 h of cultivation revealed that, under all conditions analyzed, the mutant strain exhibited lower fluorescence intensity and fewer granules per cell compared to the wild-type strain (Figures 4A–F).

Under +N conditions (20 mM), the number of granules per cell was low and did not differ statistically between strains (Figure 4G). Most cells of *H. rubrisubalbicans* M1 and hr0242:Tn5 contained one to two granules, reflecting a metabolic state in which nitrogen availability suppresses PHB accumulation. Nonetheless, *H. rubrisubalbicans* M1 exhibited higher cell density and a slightly greater proportion of cells containing two granules.

Under nitrogen limitation (−N, 5 mM), a marked and statistically significant increase (*p* ≤ 0.0001) in the number of granules per cell was observed in the wild-type strain (Figure 4G). At 12 h, many cells contained three to five cytoplasmic inclusions, and at 24 h the median remained high, with a slight reduction, indicating the onset of polymer mobilization. In contrast, the mutant strain predominantly contained cells with one or two granules, even under nitrogen limitation, and this difference was significant at both time points (*p* ≤ 0.0001).

These results indicate that PHB accumulation in *H. rubrisubalbicans* M1 is favored by nitrogen limitation and that deletion of the *Hrubri_0242* gene is associated with a reduction in both the number and distribution of intracellular granules.

Based on these morphological findings and the observed differences in accumulation patterns between strains, the next step was to assess whether these phenotypic variations correlate with changes in the transcriptional levels of genes involved in PHB metabolism, including those associated with polymer synthesis, mobilization, and regulation.

### 3.6 Expression profile of *Hrubri_0242* under different nitrogen conditions

**FIGURE 5:**
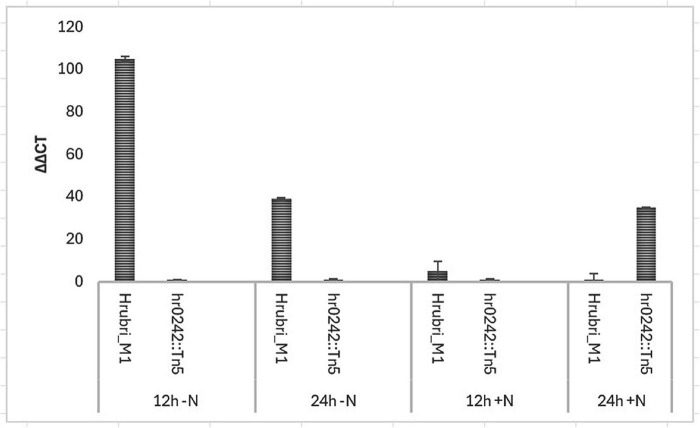
Relative expression levels of the *Hrubri_0242* gene in *Herbaspirillum rubrisubalbicans* M1 (*H. rubri*_M1) and the mutant strain hr0242:Tn5 under different nitrogen conditions and growth times. Gene expression was quantified by RT-qPCR and calculated using the ΔΔCT method. Cultures were analyzed after 12 and 24 h of growth under nitrogen-limited (–N; 5 mM) and nitrogen-replete (+N; 20 mM) conditions. The wild-type strain showed markedly higher expression under nitrogen limitation, particularly at 12 h, whereas the mutant exhibited strongly reduced transcription under –N and increased expression at 24 h under +N conditions, indicating altered regulation of nitrogen-responsive gene expression. Error bars indicate the standard deviation of three technical replicates.

The transcriptional profile of *Hrubri_0242* was evaluated by RT-qPCR in the wild-type *H. rubrisubalbicans* M1 strain and in the hr0242:Tn5 mutant under nitrogen-limiting (−N, 5 mM NH_4_Cl) and nitrogen-replete (+N, 20 mM NH_4_Cl) conditions at 12 and 24 h (Figure 5).

At 12 h under nitrogen limitation (12 h −N), the wild-type strain exhibited strong induction of *Hrubri_0242*, with ΔΔCt values of approximately 105, whereas the mutant strain showed values close to zero.

At 24 h under -N conditions, ΔΔCt values in the wild type decreased to approximately 40, while the mutant continued to present values near zero.

Under nitrogen-replete conditions (+N), ΔΔCt values were markedly lower in the wild-type strain. At 12 h +N, the wild type showed ΔΔCt values around 5, whereas the mutant exhibited values close to zero. At 24 h +N, the wild-type strain maintained low ΔΔCt values (∼2–3), while the mutant displayed increased ΔΔCt values of approximately 35.

These results indicate that *Hrubri_0242* transcription is strongly responsive to nitrogen limitation in the wild-type strain, particularly at 12 h, whereas the mutant exhibits an altered temporal expression pattern.

### 3.7 Gene expression associated with PHB metabolism

Gene expression analysis by RT-qPCR was performed to evaluate the effect of *Hrubri_0242* interruption on the regulation of genes involved in polyhydroxybutyrate (PHB) synthesis, stabilization, and degradation in *H. rubrisubalbicans* M1. The genes analyzed included *phaC1* (PHB synthase), *phaP1* and *phaP2* (phasin proteins associated with PHB granules), *phaZ1* and *phaZ2* (PHB depolymerases), and *phaR* (transcriptional regulator). Relative expression levels were determined at 12 and 24 h under high (+N, 20 mM NH_4_Cl) and low (−N, 5 mM NH_4_Cl) nitrogen conditions, using the 16S rRNA gene as an internal reference.

**FIGURE 6:**
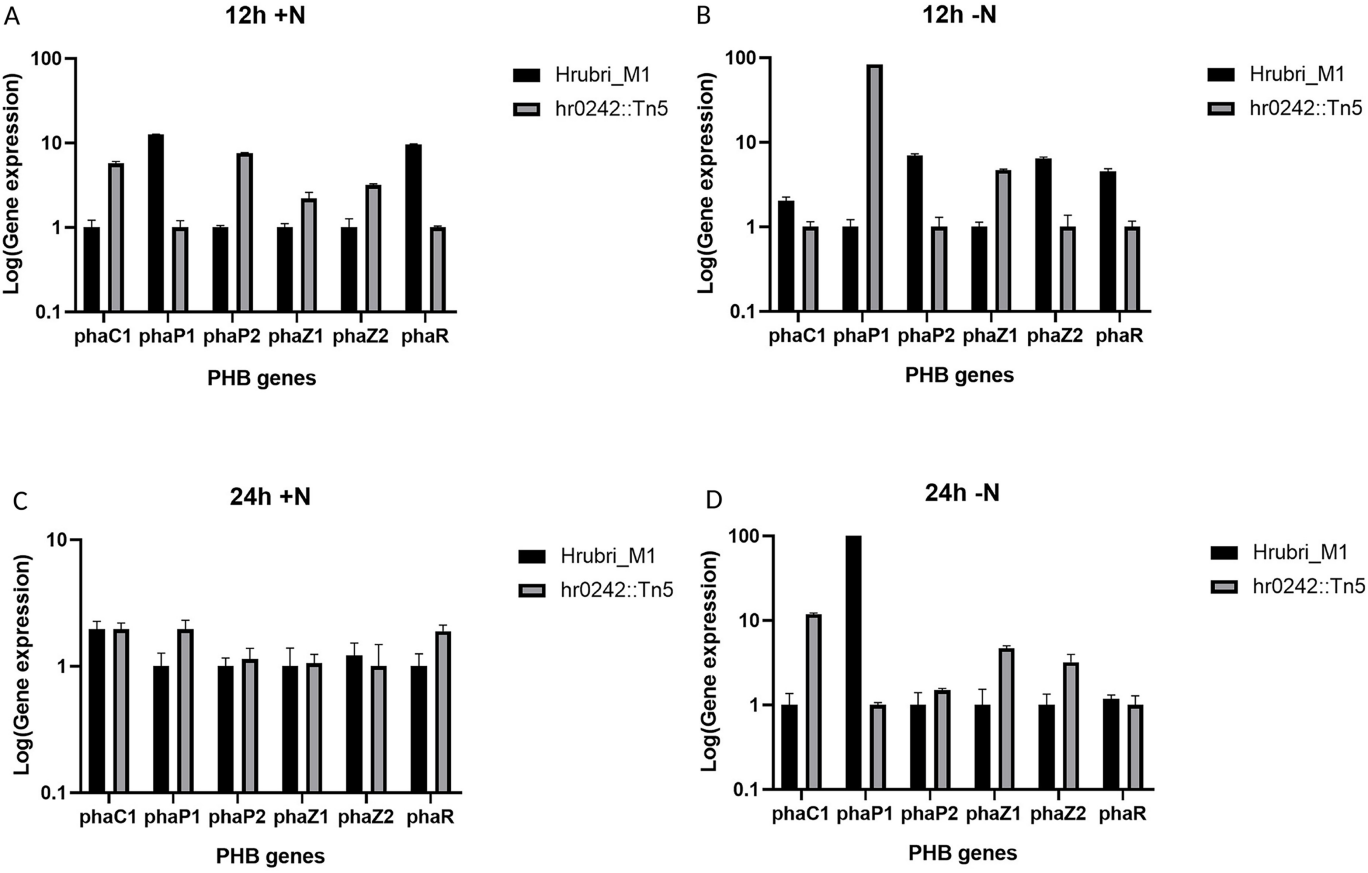
Relative expression of genes associated with polyhydroxybutyrate (PHB) metabolism in *H. rubrisubalbicans* M1 and the mutant strain hr0242:Tn5 under different nutritional conditions. Transcript levels were determined by RT-qPCR and normalized to the 16S rRNA gene. Relative expression values were calculated using the wild-type strain (*Hrubri_*M1, +N, 12 h) as the reference condition. Comparisons were performed between wild-type and mutant strains for each experimental condition. **(A)** 12 h +N. **(B)** 12 h –N. **(C)** 24 h +N. **(D)** 24 h –N. Analyzed genes include *phaC1* (PHB synthase), *phaP1* and *phaP2* (phasin proteins for granule stabilization), *phaZ1* and *phaZ2* (depolymerases), and *phaR* (transcriptional regulator). Bars represent the mean of three technical replicates; error bars indicate variability among technical measurements. As only a single biological replicate was available, no statistical analysis was performed, and the data are presented solely as qualitative support for observed expression trends. The mutant strain hr0242:Tn5 showed overexpression of *phaC1* and *phaZ1/2* under nitrogen-sufficient conditions (+N) and a delayed response under nitrogen limitation (–N), whereas the wild-type strain displayed coordinated induction of *phaP1* and *phaP2* during nitrogen starvation, indicating *Hrubri_0242*-dependent regulation of PHB synthesis and degradation.

Expression of *phaC1* showed marked differences between strains and conditions (Figure 6). At 12 h +N (Figure 6A), the mutant hr0242:Tn5 exhibited levels approximately six times higher than the wild type, indicating premature activation of the gene under non-inducing conditions. Under nitrogen limitation (12 h −N; Figure 6B), *phaC1* expression was higher in the wild-type strain, consistent with typical induction under nutritional stress (Mifune et al., 2010). At 24 h −N (Figure 6D), the mutant again showed higher expression, about 13-fold above the wild type, suggesting loss of temporal control dependent on *Hrubri_0242*.

The *phaP1* gene, encoding the main phasin involved in PHB granule stabilization, was significantly more expressed in the wild-type strain at 12 h +N (Figure 6A), whereas the mutant exhibited levels approximately 12-fold lower. At 24 h −N (Figure 6D), *phaP1* was strongly induced in the wild type (∼170-fold), whereas expression was undetectable in the mutant, indicating impaired polymer accumulation and stabilization.

Expression of *phaP2* followed a distinct pattern. At 12 h +N (Figure 6A), the mutant exhibited expression ∼7-fold higher than the wild type, but levels equalized at 24 h +N (Figure 6C). Under nitrogen limitation (12 h −N; Figure 6B), the wild-type strain showed strong induction, whereas the mutant maintained sustained but lower expression, suggesting a compensatory role of *phaP2*, albeit less efficient than *phaP1*.

Depolymerase genes *phaZ1* and *phaZ2* also exhibited contrasting profiles. At 12 h +N (Figure 6A), *phaZ1* was slightly more expressed in the mutant, a difference that increased at 12 h −N (Figure 6B), reaching values ∼4-fold higher than the wild type. *phaZ2* showed higher expression in the wild-type strain at 12 h −N (∼4-fold), indicating a greater contribution to PHB mobilization under nutritional stress. At 24 h −N (Figure 6D), both depolymerases were strongly induced in the mutant (4.5-fold for *phaZ1* and 3-fold for *phaZ2* relative to the parental strain), evidencing delayed polymer degradation.

The transcriptional regulator *phaR* maintained stable expression in the wild type under all tested conditions. In contrast, the mutant displayed significant reduction of *phaR* at 12 h +N (Figure 6A), possibly reflecting dysregulation of the cascade controlling phasin expression and the balance between PHB synthesis and degradation.

These results demonstrate that interruption of *Hrubri_0242* alters the expression dynamics of key genes in PHB metabolism, resulting in early induction of synthesis and degradation genes under non-limiting conditions and delayed response under nitrogen stress. This pattern is consistent with the loss of temporal coordination observed in polymer accumulation and consumption data, suggesting that *Hrubri_0242* functions as a regulatory element integrating PHB synthesis, stabilization, and mobilization (Mifune et al., 2010; Jarrell and McBride, 2008)

## 4 Discussion

Previous studies have demonstrated that deletion of asnC in *Pseudomonas aeruginosa* PGPR2 not only increases motility but also enhances the strain’s ability to colonize plant roots and to form biofilms, effects that were shown to be accompanied by a global reprogramming of gene expression involving hundreds of genes associated with metabolism, signaling pathways, adhesion mechanisms, and stress responses (Sivakumar et al., 2019, 2020). These observations indicate that, in this rhizosphere-associated bacterium, AsnC acts predominantly as a repressive regulator of functions related to adaptation to the root environment, such that its deletion facilitates colonization-oriented strategies rather than impairing them. In contrast, the mutation of *Hrubri_0242* in *H. rubrisubalbicans* M1 produced a significant reduction in motility relative to the wild type (Figure 3B), clearly demonstrating that despite functional homology to AsnC family regulators, the biological function of this regulator differs substantially between taxa. Because swarming motility relies on flagellar rotation and the proper synthesis and assembly of surface appendages such as type I pili, which are essential for movement and initial surface interaction, the reduced motility observed in the hr0242:Tn5 mutant suggests an impact on the pathways governing the production, integrity, or arrangement of these structures (Kearns, 2010).

### 4.1 Temporal dynamics of PHB accumulation and consumption

In addition to the observed effects on motility and biofilm production, PHB production may also influence the competitive ability of *H. rubrisubalbicans* M1 during sorghum colonization. PHB is stored by bacteria as insoluble intracellular granules, coated with proteins representing 0.5%–2% of the total granule weight (Grage et al., 2009). Initially, lipids were suggested to be present on the granule surface (Beeby et al., 2012), but subsequent studies indicated a predominantly proteinaceous composition (Koskimäki et al., 2015). PHB-producing bacteria colonize plant roots more efficiently, with the expression of PHB synthesis and degradation genes observed during colonization of grasses by *H. seropedicae* SmR1 (Alves et al., 2016; Silveira Alves et al., 2019; Pankievicz et al., 2016).

Recent studies further support that, in addition to facilitating plant interaction, PHB contributes to protection under abiotic and competitive stress conditions. In *Methylobacterium extorquens* associated with *Pinus*, production of methyl-esterified 3-hydroxybutyrate dimers and trimers (ME-3HB) conferred protection to mutants deficient in stress response genes (Koskimäki et al., 2015). Similarly, in *P. fluorescens* FR1, PHB was essential for plant adaptation under unfavorable conditions (Stritzler et al., 2018). Moreover, intracellular PHB accumulation can favor bacterial infection and differentiation during plant colonization (Lodwig et al., 2005).

Although *H. rubrisubalbicans* M1 is recognized as an endophytic and plant-associated bacterium capable of colonizing grass roots, its PHB-producing capacity had not been previously reported. Considering that this biopolymer may contribute to bacterial adaptation and symbiosis, we quantified PHB by gas chromatography (GC-FID) in *H. rubrisubalbicans* M1 and the hr0242:Tn5 mutant. Analysis of the mutant is justified as *Hrubri_0242* is a transcriptional regulator belonging to a cluster associated with PHB metabolism, which includes genes such as acyl-CoA dehydrogenase (*Hrubri_0238*), 3-hydroxyacyl-CoA dehydrogenase (*Hrubri_0239*), acetyl-CoA acetyltransferase (*phbA*; *Hrubri_0240*), and enoyl-CoA hydratase (*Hrubri_0241*). These enzymes are fundamental in PHA metabolism, linking fatty acid β-oxidation to the generation of precursors such as 3-hydroxyacyl-CoA, essential for biopolymer synthesis (Pankievicz et al., 2016).

Quantitative analyses by GC-FID revealed a dynamic pattern of PHB accumulation and consumption during cell growth, reflecting metabolic adjustments associated with nutrient availability. The wild-type strain showed progressive PHB content increase up to 12 h of cultivation, followed by a sharp decrease between 12 and 24 h, consistent with polymer consumption after carbon or nitrogen source depletion. In contrast, the hr0242:Tn5 mutant maintained relatively constant PHB levels over time, without displaying the same accumulation and subsequent consumption profile observed in the wild type. This stable PHB pattern suggests that *Hrubri_0242* plays a relevant role in regulating genes involved in polymer biosynthesis and degradation, integrating a mechanism associated with the cellular physiological response to nutrient limitation and modulating the transition between PHB accumulation and consumption.

Both the wild-type and mutant strains produced PHB under the two tested nitrogen conditions: 20 mM (+N) and 5 mM (−N). Under +N, production started between 6 and 8 h, peaking at 12 h (exponential phase), followed by progressive consumption during the stationary phase; under −N, accumulation was more prolonged and the decrease in PHB levels was less pronounced. After PHB consumption began (∼12 h), a reduction in cell viability was observed, possibly associated with carbon source depletion, with PHB acting as a survival energy substrate. PHB reserves were exhausted earlier in the mutant (16–18 h) than in the wild type (20–22 h), indicating that hr0242:Tn5 has a lower capacity to accumulate and maintain reserves, which may compromise survival under scarcity and reduce competitive efficiency during colonization (Alves et al., 2016; Lee et al., 1995).

The lower PHB production in the mutant strain can be attributed to alteration of the transcriptional regulator *Hrubri_0242*, which plays a crucial role in activating genes linked to polymer metabolism. Enzymes such as acyl-CoA dehydrogenase, enoyl-CoA hydratase, 3-hydroxyacyl-CoA dehydrogenase, and acetyl-CoA acetyltransferase are essential for providing precursors for PHB synthesis. Thus, the mutation appears to negatively impact the expression or functionality of these enzymes, reducing metabolic flux between fatty acid β-oxidation and biopolymer production. This scenario underscores the importance of *Hrubri_0242* in integrating and regulating lipid metabolism with PHB synthesis, enabling efficient conversion of fatty acids into critical intermediates, such as 3-hydroxyacyl-CoA.

Fluorescence microscopy, used to confirm the presence of intracellular granules, corroborated the GC-FID results. Two experimental time points were analyzed — 12 and 24 h — representing accumulation and consumption of PHB. Images revealed intracellular granules consistent with PHB in both strains, especially under nitrogen limitation, but with lower intensity and frequency in the hr0242:Tn5 mutant. This methodological agreement reinforces the interpretation that *Hrubri_0242* is determinant for regulating PHB metabolism and, indirectly, for bacterial adaptation under metabolic stress conditions, with implications for symbiotic and competitive competence (Alves et al., 2016; van Gemerden, 1968; Pötter and Steinbüchel, 2005).

### 4.2 Underlying mechanisms: dysregulation of the PHB pathway in the *Hrubri_0242* mutant

To investigate the role of the transcriptional regulator *Hrubri_0242* in PHB metabolism, we analyzed gene expression in the wild-type and mutant strains of *H. rubrisubalbicans* M1 under high (+N) and low (−N) nitrogen availability. In the wild-type strain, *Hrubri_0242* showed higher transcription levels under nitrogen-limiting conditions, suggesting an adaptive response to nutritional stress. In contrast, the mutant strain exhibited compromised expression under these conditions, indicating that the mutation impairs the ability to respond to nutrient limitation. Interestingly, after 24 h under +N conditions, the hr0242:Tn5 mutant showed elevated expression levels, suggesting potential alterations in regulatory mechanisms.

We also evaluated the expression of genes related to PHB synthesis, including those encoding proteins essential for this metabolic pathway. Key genes include the phasins (*phaP1* and *phaP2*), responsible for PHB granule stabilization; the synthase (*phaC1*), central for polymerization; the depolymerases (*phaZ1* and *phaZ2*), which catalyze biopolymer degradation; and the transcriptional regulator *phaR* (also known as *phbF*), which modulates intracellular PHB levels (O’Toole et al., 2000; Rehm and Steinbüchel, 1999). These proteins act in an integrated manner to regulate PHB accumulation, synthesis, and degradation, being fundamental for PHB metabolism.

The gene *phaR* is an essential transcriptional regulator controlling PHB metabolism in various bacterial species, including *Bradyrhizobium diazoefficiens* and *H. seropedicae*. In *B. diazoefficiens*, *phaR* regulates PHB production in response to nitrogen limitation, balancing energy requirements for nitrogen fixation and preventing metabolic waste (Quelas et al., 2016). Moreover, *phaR* modulates the expression of genes such as *phaC1* and *phaP1*, repressing PHB synthesis and promoting its degradation according to intracellular polymer levels (Costa et al., 2018). The regulation of phasins by *phaR* is exemplified in *Ralstonia eutropha* H16, where *phaR* represses the *phaP* promoter. This regulator binds directly to specific promoter regions, including the transcription start site and adjacent sequences containing 12-base repeats. The mechanism is dynamic and varies across different stages of PHA biosynthesis (Pötter et al., 2002).

At an initial stage, when conditions are not favorable for PHB production or PHA biosynthesis is limited, *phaR* binds the *phaP* promoter, repressing transcription. As the cell synthesizes PHA and forms PHB granules, *phaR* binds directly to the granules, relieving repression and allowing *phaP* transcription activation, facilitating polymer accumulation. When PHB accumulation reaches high levels, *phaR* dissociates from the granules and resumes its repressor function, controlling polymer synthesis and stabilization. This repression–activation cycle exemplifies adaptive *phaR* regulation in response to cellular metabolic needs and environmental conditions (Pötter et al., 2002; Maehara et al., 2002; Kadowaki et al., 2011).

Gene expression analysis revealed that *phaC1* is expressed, but *phaP1* is not. As a compensatory mechanism, *phaP2* was activated; however, its efficiency in stabilizing PHB granules is lower than that of *phaP1* (Alves et al., 2016). These findings suggest that the mutation in hr0242:Tn5 may impair *PhaP1* expression or regulation, consistent with the lack of PHB detection in gas chromatography assays. Studies on *PhaP1* and *PhaP2* activity have shown that, even when *PhaP1* is expressed, the absence of intracellular PHB may result from polymer synthesis failure or granule stabilization dysfunction. A similar reasoning applies to *PhaP2*, whose functional deficiency contributes to PHB non-accumulation in the mutant strain (Alves et al., 2016).

In *H. seropedicae*, two additional PHB metabolism genes, *phaP2* (*Hsero_4759*) and *phaZ2* (*Hsero_0639*), are strongly regulated under nitrogen limitation, encoding a phasin and a putative PHB depolymerase, respectively. Although overexpressed at low ammonium concentrations, these genes appear to play secondary roles, complementing predominant proteins under normal growth conditions (Bonato et al., 2024). In *H. seropedicae*, *phaP1* (*Hsero_1639*) is the predominant phasin in PHB granules, while *phaP2* levels are significantly lower but can functionally substitute *phaP1* in Δ*phaP1* mutants, acting as an alternative granule-stabilizing protein (Tirapelle et al., 2013; Alves et al., 2016). Under nitrogen limitation, *phaP2* was strongly induced, suggesting an adaptive role during nutritional stress, though its functional relevance requires further investigation (Bonato et al., 2024).

Polyhydroxybutyrate mobilization occurred primarily via the constitutively expressed PHB depolymerase *phaZ1* (*Hsero_1622*), while *PhaZ2* acted complementarily under specific nutrient-restricted conditions (Bonato et al., 2024). The differential regulation observed in *H. seropedicae* provides an interpretative model for understanding the effects of *Hrubri_0242* deletion in *H. rubrisubalbicans* M1, particularly regarding the balance between phasins and depolymerases.

In the wild-type *H. rubrisubalbicans* M1, PHB production reflected a coordinated balance between synthesis, stabilization, and degradation. Under +N conditions (12 h), high biopolymer synthesis resulted from elevated *phaC1* expression, associated with *phaP1* expression, essential for efficient polymer accumulation. Concurrently, low *phaZ1* and *phaZ2* expression reduced polymer mobilization, favoring storage. Under -N conditions, coordinated regulation of *phaC1*, *phaZ1*, and *phaZ2* demonstrated finely tuned control between synthesis and degradation, consistent with the cell’s nutritional state.

In the hr0242:Tn5 mutant, this balance was disrupted. Despite high *phaC1* expression indicating strong synthetic potential, low *phaP1* expression impaired polymer accumulation. As a compensatory mechanism, *phaP2* was activated; however, its capacity to stabilize granules is inferior to *phaP1*, and the overexpression of *phaZ1* and *phaZ2* enhanced PHB degradation, resulting in limited production, particularly under +N. Under −N, the concurrent high expression of *phaC1* and *phaZ1*, along with reduced *phaZ2* expression, created an imbalanced metabolism where synthesized PHB was rapidly degraded, limiting storage efficiency. Studies in other bacteria, such as *Cupriavidus necator* and *R. eutropha* H16, corroborate the importance of depolymerases in PHB metabolism (Pötter et al., 2002; Tirapelle et al., 2013). *PhaZ1* preferentially degrades larger granules and is essential under nutrient limitation, while *PhaZ2* exhibits slower kinetics, regulating granule size and maintenance. The compensatory capacity between these enzymes, along with adaptive *phaP2* regulation under nutritional stress, highlights the dynamic and integrated role of these genes in intracellular homeostasis and PHB metabolism, both under nutrient abundance and scarcity (Bonato et al., 2024).

Overall, these results demonstrate that dysregulation of PHB synthesis–degradation balance in the mutant strain significantly impairs polymer storage capacity. In contrast, the wild-type strain maintains coordinated regulation of these genes, enabling more efficient metabolism aligned with physiological needs and environmental conditions. This coordination suggests that *Hrubri_0242* acts as a central regulatory element, connecting PHB metabolism to essential cellular processes for adaptation and plant host interaction.

Collectively, the findings indicate that this transcriptional regulator plays a crucial role in coordinating physiological and metabolic pathways in *H. rubrisubalbicans* M1. Its deletion impairs not only motility and adhesion—key factors for plant colonization—but also destabilizes PHB metabolism, an essential component for adaptation and survival under nutrient limitation. The abnormal regulation of genes involved in PHB synthesis, stabilization, and degradation indicates that *Hrubri_0242* functions as an integrative link between energy metabolism and adaptive responses associated with motility and biofilm formation. Consequently, loss of this regulatory control reduces the bacterium’s symbiotic and competitive competence, limiting efficiency in *S. bicolor* colonization. These findings expand our understanding of AsnC/Lrp regulators in endophytic bacteria and highlight their role as key modulators of pathways linking metabolism, cellular behavior, and plant–bacteria interaction.

## Data Availability

The sequencing data are available in the ArrayExpress database at www.ebi.ac.uk/arrayexpress under accession number E-MTAB-3538.

## Footnotes

1
https://blast.ncbi.nlm.nih.gov/

2
https://www.genome.jp/tools-bin/clustalw

3
https://www.cathdb.info/

4
https://www.kegg.jp/kegg/

5
https://www.sanger.ac.uk/
