# Supplementary material for: Characterization of an AsnC/Lrp-family transcriptional regulator in Herbaspirillum rubrisubalbicans M1: linking plant interaction, nitrogen response and PHB metabolism
Source: Front Microbiol. 2026 Jun 4;17:1792436. doi: 10.3389/fmicb.2026.1792436 (PMC13275390; doi:10.3389/fmicb.2026.1792436)
Supplement: Supplementary file 1 [file Table_1.docx]

Supplementary material

**Supplementary Table S1-** Primers used for quantitative PCR (qPCR) analysis of genes involved in polyhydroxyalkanoate (PHA) metabolism in *Herbaspirillum rubrisubalbicans* M1. The table lists the target gene, locus tag, and the corresponding forward and reverse primer sequences (5′–3′) used for amplification. Genes include those encoding phasin proteins (*phaP1*, *phaP2*), PHA depolymerases (*phaZ1*, *phaZ2*), PHA synthase (*phaC1*), and the transcriptional regulator *phaR* (*phbF*).

| Gene | Locus tag | Forward primer (5′–3′) | Reverse primer (5′–3′) |
| --- | --- | --- | --- |
| *Hrubri_0242* | | GGAAATCGTCGAGTGCTACAA | CAGCAGTCGCTCCATCAAT |
| *phaP1* | *Hrubri_1525* | GATCCTTGGCTGCGAACA | CTGGTGGACCTGAATCTGAAG |
| *phaP2* | *Hrubri_4717* | CTACGAGCAACTGTCCAAGAA | CAGCAGTGAACTGTTTGGTTG |
| *phaZ1* | *Hrubri_0609* | GGCGCAGATCAAGGATTTCTA | GCGGACCATAGCTCAACTT |
| *phaZ2* | *Hrubri_1511* | GCCAGATTGAAGTCCTGGAATA | CCGCAAGTTCTACGACGAATA |
| *phaC1* | *Hrubri_3020* | AGGAAATGAGGAACACCGTATG | CAAGTTCTACATCCTCGACCTG |
| *phaR (phbF)* | *Hrubri_3018* | ACCACGGTGAATTCCTCATT | AACCGCCGTCTTTACGATAC |


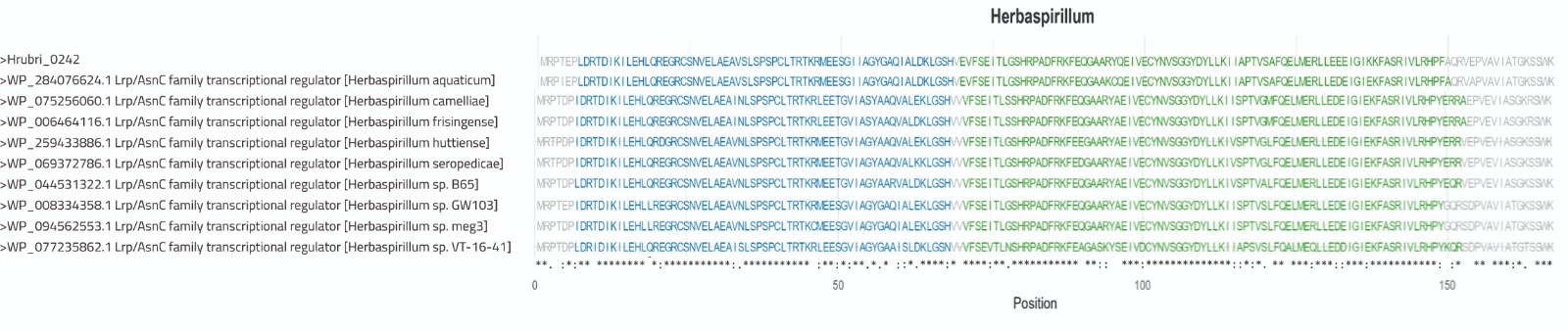


**Supplementary Figure S1 –** Multiple sequence alignment of the *Hrubri_0242* protein from *Herbaspirillum rubrisubalbicans* M1 and homologous proteins from *Herbaspirillum* species.

Sequences were selected based on BLASTp searches against the NCBI RefSeq database and filtered according to sequence identity and coverage criteria, as described in the Materials and Methods. The alignment was generated using ClustalW to assess intra-genus conservation of the Lrp/AsnC family transcriptional regulator. Conserved residues are indicated by an asterisk (*), conservative substitutions by a colon (:), and semi-conservative substitutions by a dot (.). The helix–turn–helix (HTH) DNA-binding domain is indicated in blue, and the Lrp/AsnC regulatory domain is indicated in green.


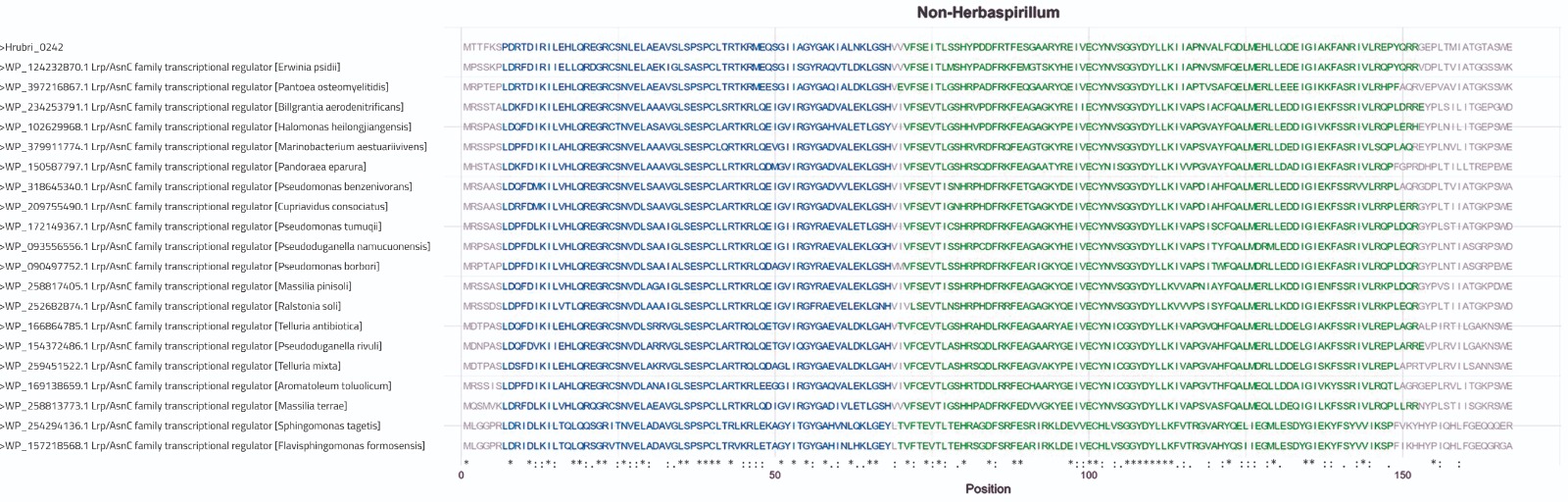


**Supplementary Figure S2 –** Multiple sequence alignment of the *Hrubri_0242* protein from *Herbaspirillum rubrisubalbicans* M1 and homologous proteins from phylogenetically distinct bacterial genera.

Sequences were selected based on BLASTp searches against the NCBI RefSeq database and filtered according to sequence identity and coverage criteria, as described in the Materials and Methods. The alignment was generated using ClustalW to evaluate conservation of the Lrp/AsnC family transcriptional regulator across taxonomically diverse bacteria. Conserved residues are indicated by an asterisk (*), conservative substitutions by a colon (:), and semi-conservative substitutions by a dot (.). The helix–turn–helix (HTH) DNA-binding domain is indicated in blue, and the Lrp/AsnC regulatory domain is indicated in green.

**Supplementary Table S2.** Predicted conserved domains in *Hrubri_0242* and homologous proteins from *Herbaspirillum* species identified using the InterPro database. The table shows protein length (Seq_length) and the predicted start and end positions of conserved domains characteristic of the AsnC/Lrp family of transcriptional regulators. These include the Lrp domain and the helix–turn–helix (HTH) DNA-binding domain, as well as the AsnC regulatory domain. Sequence identifiers correspond to protein accession numbers from NCBI, and the domain coordinates indicate their relative positions within each protein sequence. The conserved organization of these domains across the analyzed sequences supports the classification of *Hrubri_0242* as a member of the AsnC/Lrp transcriptional regulator family in *Herbaspirillum*.

| **Seq** | **Seq_length** | **Lrp** | | **HTH** | | **AsnC** | |
| --- | --- | --- | --- | --- | --- | --- | --- |
|  |  | Start | End | Start | End | Start | End |
| [Hrubri_0242](https://www.ebi.ac.uk/interpro/result/InterProScan/iprscan5-R20260311-175145-0205-41209420-p1m/internal-1773251500935-2-1/) | 167 | 6 | 155 | 7 | 68 | 70 | 149 |
| [WP_044531322.1](https://www.ebi.ac.uk/interpro/result/InterProScan/iprscan5-R20260311-175145-0205-41209420-p1m/internal-1773251500935-2-2/) | 167 | 6 | 156 | 7 | 68 | 70 | 150 |
| [WP_284076624.1](https://www.ebi.ac.uk/interpro/result/InterProScan/iprscan5-R20260311-175145-0205-41209420-p1m/internal-1773251500935-2-3/) | 167 | 5 | 156 | 7 | 68 | 71 | 152 |
| [WP_008334358.1](https://www.ebi.ac.uk/interpro/result/InterProScan/iprscan5-R20260311-175145-0205-41209420-p1m/internal-1773251500935-2-4/) | 167 | 5 | 156 | 7 | 68 | 71 | 152 |
| [WP_075256060.1](https://www.ebi.ac.uk/interpro/result/InterProScan/iprscan5-R20260311-175145-0205-41209420-p1m/internal-1773251500935-2-5/) | 167 | 5 | 156 | 7 | 68 | 71 | 153 |
| [WP_094562553.1](https://www.ebi.ac.uk/interpro/result/InterProScan/iprscan5-R20260311-175145-0205-41209420-p1m/internal-1773251500935-2-6/) | 167 | 5 | 155 | 7 | 68 | 71 | 152 |
| [WP_006464116.1](https://www.ebi.ac.uk/interpro/result/InterProScan/iprscan5-R20260311-175145-0205-41209420-p1m/internal-1773251500935-2-7/) | 167 | 6 | 154 | 7 | 68 | 71 | 149 |
| [WP_069372786.1](https://www.ebi.ac.uk/interpro/result/InterProScan/iprscan5-R20260311-175145-0205-41209420-p1m/internal-1773251500935-2-8/) | 167 | 5 | 156 | 7 | 68 | 71 | 153 |
| [WP_259433886.1](https://www.ebi.ac.uk/interpro/result/InterProScan/iprscan5-R20260311-175145-0205-41209420-p1m/internal-1773251500935-2-9/) | 167 | 5 | 156 | 7 | 68 | 71 | 152 |
| [WP_077235862.1](https://www.ebi.ac.uk/interpro/result/InterProScan/iprscan5-R20260311-175145-0205-41209420-p1m/internal-1773251500935-2-10/) | 167 | 5 | 154 | 7 | 68 | 71 | 149 |

**Supplementary Table S3.** Comparative analysis of conserved domains in *Hrubri_0242*from *Herbaspirillum rubrisubalbicans* and homologous proteins from diverse bacterial genera. Protein sequences were analyzed using the InterPro database to identify conserved domains characteristic of the AsnC/Lrp family of transcriptional regulators. The table shows the total protein length (Seq_length) and the predicted start and end positions of the Lrp domain, the helix–turn–helix (HTH) DNA-binding motif, and the AsnC regulatory domain within each sequence. Protein accession numbers correspond to NCBI entries and include homologs from multiple bacterial taxa. The conserved domain architecture across phylogenetically distinct bacteria indicates strong structural conservation of the Lrp/AsnC regulatory module and supports the classification of *Hrubri_0242* as a member of this transcriptional regulator family.

| Seq | Seq_length | Lrp | | HTH | | AsnC | |
| --- | --- | --- | --- | --- | --- | --- | --- |
|  |  | Start | End | Start | End | Start | End |
| *Hrubri_0242* | 167 | 6 | 155 | 7 | 68 | 70 | 149 |
| WP_209755490.1 | 167 | 5 | 155 | 7 | 68 | 71 | 152 |
| WP_172149367.1 | 167 | 5 | 156 | 7 | 68 | 71 | 152 |
| WP_258817405.1 | 167 | 4 | 149 | 7 | 68 | 71 | 148 |
| WP_093556556.1 | 167 | 4 | 156 | 7 | 68 | 71 | 152 |
| WP_259451522.1 | 167 | 5 | 156 | 7 | 68 | 71 | 153 |
| WP_252682874.1 | 167 | 5 | 156 | 7 | 68 | 72 | 152 |
| WP_157218568.1 | 167 | 6 | 149 | 7 | 68 | 70 | 148 |
| WP_397216867.1 | 167 | 5 | 153 | 7 | 68 | 71 | 152 |
| WP_102629968.1 | 167 | 4 | 156 | 7 | 68 | 71 | 152 |
| WP_166864785.1 | 167 | 5 | 156 | 7 | 68 | 70 | 152 |
| WP_379911774.1 | 167 | 5 | 156 | 7 | 68 | 71 | 153 |
| WP_169138659.1 | 167 | 5 | 156 | 7 | 68 | 71 | 152 |
| WP_258813773.1 | 167 | 5 | 156 | 7 | 68 | 71 | 149 |
| WP_318645340.1 | 167 | 5 | 156 | 7 | 68 | 71 | 152 |
| WP_090497752.1 | 167 | 5 | 156 | 7 | 69 | 71 | 152 |
| WP_154372486.1 | 167 | 6 | 149 | 7 | 68 | 71 | 149 |
| WP_254294136.1 | 167 | 6 | 156 | 7 | 68 | 70 | 148 |
| WP_150587797.1 | 167 | 6 | 156 | 7 | 68 | 71 | 151 |
| WP_234253791.1 | 167 | 4 | 152 | 7 | 68 | 71 | 149 |
| WP_124232870.1 | 167 | 7 | 155 | 7 | 68 | 71 | 152 |

**Supplementary Table S4 -** Functional annotation of genes located in the genomic cluster harboring *Hrubri_0242*, as determined by *in silico* analysis using the Kyoto Encyclopedia of Genes and Genomes (KEGG) database, including predicted functions and associated metabolic pathways.

| ID_gene | Function | Metabolic Pathways |
| --- | --- | --- |
| *Hrubri_0234* | type VI secretion system secreted protein VgrG | Bacterial secretion system |
| *Hrubri_0235* | ribosome-associated heat shock protein Hsp15 | n/a |
| *Hrubri_0236* | hypothetical protein | n/a |
| *Hrubri_0237* | transcription regulator, TetR Family | n/a |
| *Hrubri_0238* | acyl-CoA dehydrogenase | n/a |
| *fadB* | 3-hydroxyacyl-CoA dehydrogenase | Fatty acid degradation |
| *phbA* | acetyl-CoA acyltransferase | Fatty acid degradation |
| *Hrubri_0241* | Enoyl-CoA hydratase/isomerase family protein | n/a |
| *Hrubri_0242* | AsnC family transcription regulator protein | n/a |
| *gabT* | 4-aminobutyrate aminotransferase | n/a |
| *Hrubri_0244* | Phosphotransferase | Lysine degradation |
| *hadL* | Dehalogenase | Chlorocyclohexane and chlorobenzene degradation |
| *dadA* | FAD dependent oxidoreductase | n/a |
